# A Self-Guided Digital Mental Health Promotion Service Targeting Young People: Protocol for a Randomized Controlled Trial

**DOI:** 10.2196/73736

**Published:** 2025-09-10

**Authors:** Sofie Have Hoffmann, Amalie Oxholm Kusier, Isabelle Pascale Mairey, Anna Paldam Folker, Lau Caspar Thygesen

**Affiliations:** 1 National Institute of Public Health University of Southern Denmark Copenhagen K Denmark

**Keywords:** mental health, adolescent, health promotion, randomized controlled trial, effectiveness study, qualitative research, cost-effectiveness

## Abstract

**Background:**

The high and increasing rate of poor mental health among young people is a matter of global concern. Experiencing poor mental health during this formative stage of life can adversely impact interpersonal relationships, academic and professional performance, and future health and well-being if not addressed early. However, only a few of those in need seek help. Research indicates that young people perceive digital mental health support as having many benefits compared to traditional face-to-face services. However, the effectiveness of self-guided digital mental health services is not well documented, and research on their cost-effectiveness is lacking. Mindhelper is Denmark’s largest open access, digital, self-guided mental health service for young people. While it does not provide direct psychological or therapeutic care, it offers practical strategies and tools to promote well-being and address a broad spectrum of mental health challenges, from everyday stress to more complex issues. Despite its widespread use, the effectiveness of Mindhelper has not been evaluated.

**Objective:**

This trial aims to evaluate the effectiveness of building on the results of our feasibility study. We will assess Mindhelper’s impact on mental health and well-being, psychological functioning, intentions of help seeking, and body appreciation among people aged between 15 and 25 years and provide insights into the service’s cost-effectiveness.

**Methods:**

We will recruit 4910 people aged between 15 and 25 years via social media and randomized and allocated to an intervention group (receiving information about Mindhelper) or a control group (no information about Mindhelper). Outcomes are self-assessed and collected at baseline and 2, 6, and 12 weeks after randomization through online surveys and analyzed using the intention-to-treat approach. Qualitative interviews with intervention group participants will provide complementary insights, and a cost-effectiveness analysis will also be conducted.

**Results:**

This study was fully funded in November 2022, and the data collection started in January 2025. As of August 2025, we enrolled 2613 people. The data analysis will start after data collection concludes (by early 2026), and the results of the primary outcome are expected to be published in the second half of 2026.

**Conclusions:**

This study will deliver crucial evidence on the effectiveness of self-guided digital mental health promotion targeting young people. If effective, this highly scalable service may contribute to combating the trend of rising mental health issues among young people and address key challenges in primary care by delivering timely, coordinated, and effective services to young individuals, potentially at a low cost.

**Trial Registration:**

ClinicalTrials.gov NCT06385457; https://clinicaltrials.gov/ct2/show/NCT06385457

**International Registered Report Identifier (IRRID):**

DERR1-10.2196/73736

## Introduction

### Background

The high and increasing rate of poor mental health in young people is a matter of global concern [1], and in Denmark, the proportion of people aged between 16 and 24 years reporting poor mental health increased from 12% in 2010 to 26% in 2023 [2,3]. If left unaddressed, poor mental health (even below the diagnostic threshold) during this formative stage of life can negatively affect interpersonal relationships, academic and professional performance, and long-term health and well-being [1,4]. Young people constitute the age group most affected by mental health problems, yet only a few of those in need seek help. Barriers to seeking help include concerns about stigma, confidentiality, difficulties navigating mental health services, and a preference for informal help [5-7].

Digital services, such as apps and websites, offer significant potential to enhance the scalability of mental health services. Research indicates that young people perceive digital mental health support as having many benefits compared to traditional face-to-face services, including greater accessibility, more interactivity, greater anonymity, and reduced stigma associated with help seeking [5,8-13]. Most digital mental health services focus on addressing symptoms after their onset, primarily targeting depression and anxiety, and digital services designed to reduce these conditions have shown small but promising effects [13-16]. However, evidence for symptoms beyond depression and anxiety remains limited, making the results more inconclusive [13].

Digital services incorporating an in-person element (eg, therapist or other health professional) are associated with greater effectiveness, better adherence, and lower dropout rates compared to fully self-guided services [13,15]. Nevertheless, self-guided services are highly scalable, as they can be delivered to a higher number of users with low additional costs. Although the effects remain mixed, self-guided smartphone apps have shown the potential for improving symptoms across various domains, particularly for depression [17].

Most studies assess the effects of an experimental service designed by researchers for testing in a controlled research setting, aimed at reducing one mental health issue, with little research on multicomponent services applied in real-world settings focusing on broader mental health problems. Samples are often drawn from narrowly defined populations, such as students from a single university or individuals with specific symptomatology, thereby reducing generalizability to real-world settings. Few large-scale randomized controlled trials (RCTs) have been conducted, as most involve fewer than 250 participants [14,17]. Furthermore, despite being widely considered as low cost, there is a lack of evidence on the cost-effectiveness of digital mental health services targeting young people [13].

Mindhelper is Denmark’s largest open access, digital, self-guided mental health service for young people. While it does not provide direct psychological or therapeutic care, it offers practical strategies and tools to promote well-being and address a broad spectrum of mental health challenges, from everyday stress to more complex mental health issues. Despite its widespread use, the effectiveness of Mindhelper has not been evaluated.

### Objectives

Building on the results of our feasibility study [18], this research will evaluate Mindhelper’s impact on mental health and well-being, psychological functioning, intentions of help seeking, and body appreciation among people aged between 15 and 25 years. In addition, it will provide insights into the service’s cost-effectiveness. We aim to recruit 4910 participants, making this the largest RCT to assess the effects of a self-guided digital mental health promotion service targeting young people.

## Methods

### Ethical Considerations

The study protocol follows the Standard Protocol Items: Recommendations for Interventional Trials checklist [19]. It was legally approved by the University of Southern Denmark (11.882) and ethically approved by the research ethics committee of the University of Southern Denmark (24-2933). Furthermore, the study was registered on ClinicalTrials.org before its initiation (NCT06385457). Before randomization, all participants are given additional information before they provide consent to participate. Participants who complete all follow-up surveys will be included in a lottery for cinema gift cards (DKK 299 [US $43]), with 200 gift cards being distributed.

### Intervention: Mindhelper

Mindhelper is a service hosted at the Centre for Digital Psychiatry in Denmark. Mindhelper’s primary service is a website [20] launched in 2016, which provides information, self-help tools, and evidence-based guidance on various mental health challenges that young people face today. This includes dealing with anxiety, loneliness, family difficulties, depression, romantic relations, eating disorders, and self-harm.

Mindhelper is different from other Danish digital mental health services in that it is the most comprehensive website containing unstructured information and tools across the whole spectrum of mental health problems and issues that young people may face in their everyday lives. Mindhelper is also unique in being integrated into the public mental health system and is free to use for everyone, self-administered, and self-guided.

Tools and information on Mindhelper are developed specifically for and with young people to enhance usability and encourage help seeking [21]. Most material is based on principles derived from cognitive behavioral theory, including mindfulness exercises and acceptance and commitment therapy–inspired self-instructions on beliefs, values, and general strategies for good mental health and well-being [22-24]. The website further serves as a national directory for local youth mental health services for further support and help.

The Mindhelper website also offers a supportive outreach service, where psychology students respond to digital letters sent by young people, supported by experienced psychologists when needed. The letters exchanged are published in an anonymized form so that other young people with similar concerns or worries may benefit from the supportive advice. Mindhelper was codeveloped between 2014 and 2016 by the Centre for Digital Psychiatry in partnership with young people and 4 Danish municipalities (Svendborg, Odense, Faaborg-Midtfyn, and Varde). The primary inspiration for Mindhelper is the Australian platform ReachOut [25].

Throughout its development and dissemination phase, Mindhelper has adopted a participatory approach, co-designing its resources with young people, parents, teachers, school counselors, youth workers, psychologists in community care, general practitioners, and youth mental health experts. More than 300 young people have participated in workshops, user panels, and content production teams. Iterative coproduction methods, based on the core principles of design thinking (understanding, defining, ideating, prototyping, and refining), have been crucial in ensuring the service remains relevant and useful to young people [26-28].

Since January 2019, Mindhelper has received permanent funding through a joint agreement between the 5 Danish regions and has been freely available and disseminated to young people and youth mental health professionals across the country. Mindhelper receives approximately 1 million visits a year [29]. Mindhelper’s primary target group is people aged between 13 and 20 years; however, it also includes adults, parents, and youth mental health professionals [29].

In 2024, Mindhelper launched their first mobile app named Ro (Ro is Danish for calm), aiming to reduce stress and increase everyday calmness. The app is free to use and may be downloaded on Android smartphones and iPhones. In this paper, the Mindhelper website and the app Ro will be referred to commonly as “Mindhelper.”

The research team was not involved in developing Mindhelper or its maintenance or promotion. Partnering with the Centre for Digital Psychiatry meant that the service tested builds on evidence-based information and is built by an expert team. Furthermore, the service is sustainable (independent of research funds) compared to most services tested, as Mindhelper was constructed and is funded and used independently of this study.

### Study Design

**Figure 1:**
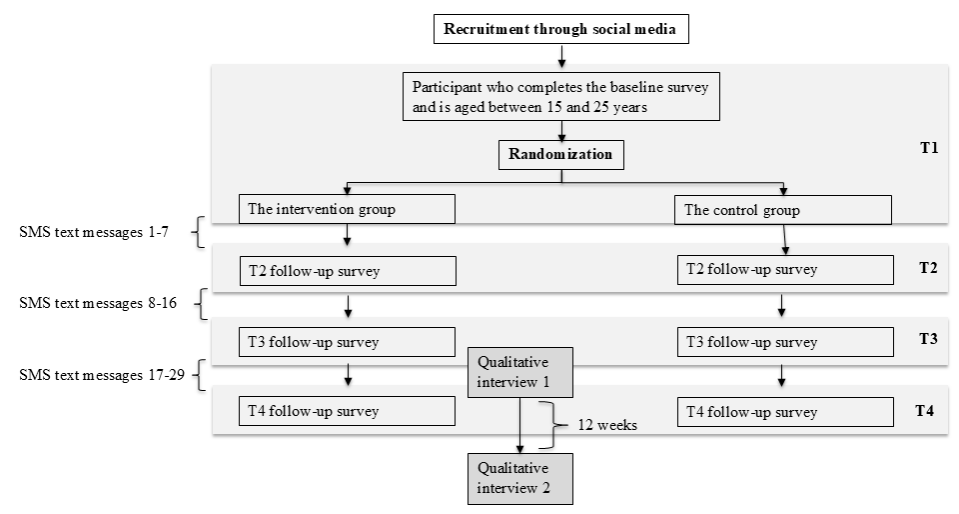
Overview of the study design.

The effects of Mindhelper will be evaluated in a 2-armed 1:1 RCT and complemented by qualitative insights from interviews with participants assigned to the intervention group. Figure 1 provides an overview of the study design.

Participants are randomized upon responding to the baseline (T1) survey, and based on their group assignment, they will receive information about Mindhelper at the end of the survey (intervention group) or be informed that they have been placed in the control group. All data are collected online (in SurveyXact [Ramboll Management Consulting]). Randomization is conducted automatically in the survey setup and is done independently of the participants’ responses at T1. There is no care provider or outcome assessor, as all measures are self-reported and collected through online questionnaires. Randomization, invitations for follow-ups, reminders to nonresponders, and distribution of information to the intervention group were all done automatically and independently of the research team.

By the end of the T1 survey, participants are asked to provide their mobile numbers, and invitations to follow-up surveys are sent by SMS text messages. Therefore, it is solely participants completing the T1 survey who can be invited to the follow-up surveys. In our feasibility study, participants preferred being contacted via SMS text messages as compared to emails, which is consistent with studies exploring how to engage young people in digital mental health interventions [18,30].

All participants aged between 15 and 25 years who complete the T1 survey will be invited to respond to follow-up surveys 2 weeks after randomization (T2), 6 weeks after randomization (T3), and 12 weeks after randomization (T4). By the end of the survey at T4, the control group will be informed about Mindhelper and directed to the website.

Our feasibility study revealed very low engagement when participants were merely provided with a link to Mindhelper at the end of the T1 survey [18]. To increase use in the intervention group, we developed an SMS text message campaign. Participants will receive 29 SMS text messages throughout the study, each offering tips and guidance on using Mindhelper. Each SMS text message links to a restricted section of the website, where participants are introduced to a specific theme or feature through brief texts and videos before being guided to the unrestricted section of the website. Multimedia Appendix 1 provides a detailed overview of the campaign themes.

The recruitment material states that the study is evaluating a mental health promotion website for young people, but does not mention Mindhelper specifically, to avoid influencing the control group’s use. Mindhelper remains publicly accessible during the study, and there is no way to ban access of participants from the control group. However, our feasibility study found that very few control group participants accessed the website, suggesting this is not a significant concern [18].

The intervention group participants may use the service as much as they like. Participants in the intervention group and control group may continue with any usual care they receive in addition to the trial. Nonresponse is minimized by sending up to 2 reminders.

At T3, participants in the intervention group will be invited to participate in 2 interviews (when responding to the survey): the 1st close to T3 and a follow-up interview approximately 12 weeks later. Further details of the qualitative part of the study are presented in the section “Qualitative Contributions.”

At the end of each survey, it is emphasized that the research team does not provide counseling or treatment and will not contact anyone based on their survey responses. Participants (in the intervention and control group) are strongly encouraged to seek help and support if they experience distress or require assistance and are directed to counseling services, including Børnetelefonen and HØRT (Danish consulting services for young people). Furthermore, in cases of acute suicidal crises, they are encouraged to contact Livslinien (a Danish service for people with suicidal thoughts) or emergency services.

### Recruitment

We aim to recruit approximately 4910 participants via Facebook [Meta Platforms, Inc], Instagram [Meta Platforms, Inc], and Reddit (the final number of participants depends on attrition at T2 as described in the Sample Size Calculation section).

The only inclusion criterion is age, with participants required to be aged between 15 and 25 years old at T1.

We aim to recruit participants in need of mental health promotion services, targeting a study population with below-average mental health and well-being status rather than a representative sample. However, as Mindhelper is a universal public health service, we have decided that all participants are eligible regardless of mental health status at T1, allowing us to assess the service’s effectiveness in the broader target population.

Our feasibility study demonstrated that Facebook and Instagram were effective platforms for recruiting young people, and the self-selection based on the framing of advertisements resulted in a study population with below-average mental health and well-being status: 49.6% (275/554) of the sample had experienced a mental illness, and of those, 49.1% (135/275) reported daily impairment in the past 12 months [18]. In addition, 64.3% (356/554) of the respondents were identified as being at risk for depression or stress based on the World Health Organization–Five Well-Being Index (WHO-5) [18].

Before initiating data collection, we defined an optimal study population composition based on the parameters available for targeted advertisements: gender and age (from user profiles) and geographical location (from IP addresses). We aimed for a sample of 70% women, as young women generally report lower mental health and well-being than young men [3]. To ensure balanced representation, we aimed for an equal distribution across ages, as unpublished data from Danes’ Health—the National Health Profile 2011 indicated no substantial age-related differences in mental health and well-being among people aged between 16 and 25 years. Similarly, because no substantial regional variation was observed in the same data for this age group, we sought to match the regional distribution of the general population in this age group.

The recruitment strategy will be monitored weekly throughout the study period and adjusted to target individuals underrepresented in the sample based on these characteristics. The ability to create multiple advertisements targeting different populations, closely monitor their real-time performance, and continuously adjust them makes online advertising a powerful tool for recruiting according to specific demographic requirements. However, as of November 6, 2023, Meta Platforms, Inc has prohibited direct advertisement to individuals aged <18 years due to European Union legislation. This regulatory change may challenge our goal of achieving equal representation across age groups, yet it remains our target.

In the feasibility study, we neither defined an optimal study population composition nor adjusted the recruitment strategy, resulting in a sample with 87.3% (488/559) women [18]. To increase male participation, we will include Reddit as a recruitment platform in this study. As of 2024, 63.6% of Reddit’s global users were male individuals [31], compared to 52% on Instagram [32] and 57% on Facebook [33]. In Denmark, 41% of Instagram users and 46% of Facebook users are male individuals [34]. While Danish user demographics for Reddit are unavailable, we assume they align with global trends.

**Figure 2:**
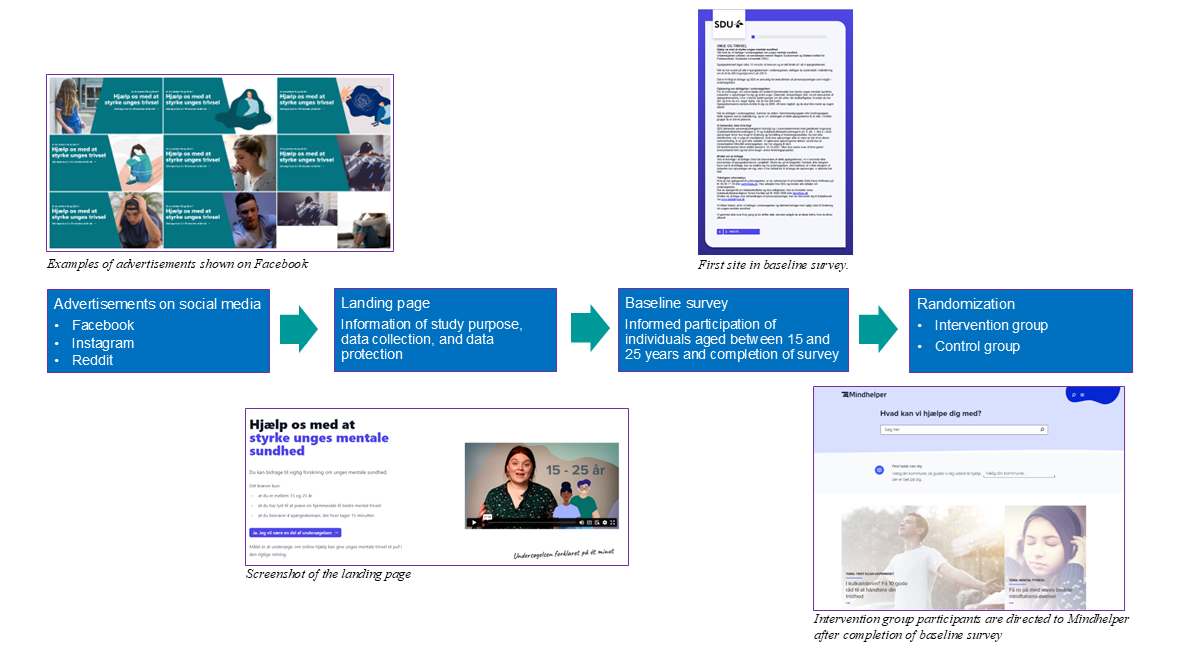
Recruitment flow from advertisement to participant.

Figure 2 illustrates the recruitment process, from viewing an advertisement on one of the platforms to becoming a study participant. From the advertisements, young people will be directed to a landing page containing information about the study design, data protection, and other details, presented with graphics and a short explainer video [35]. If they choose to participate, they will be guided to the T1 survey and provided with additional information before they provide consent to participate. The participants are informed that the study is conducted in collaboration between the region of Southern Denmark and the National Institute of Public Health, University of Southern Denmark.

At the end of the T1 survey, the intervention group will receive information about Mindhelper and will be automatically directed to Mindhelper [20], while the control group will not receive any information about Mindhelper but instead receive instructions about the next steps in the study. website

### Measures and Outcomes

#### Overview

The data are self-reported and include demographic characteristics, use of Mindhelper, and validated scales for mental health and well-being (Table 1). Though we include some measures capturing negative aspects of mental health and well-being, the primary focus in the surveys is on positive aspects rather than symptomatology.

The primary outcome is mental well-being, measured using the WHO-5, while the secondary outcome is well-being and psychological functioning assessed with the Short Warwick-Edinburgh Mental Well-being Scale (SWEMWBS). In addition, the intention of and barriers to help seeking are explorative outcomes. The impact of Mindhelper will also be evaluated on body appreciation, and an economic effectiveness analysis will be conducted.

Where no suitable scale existed for specific measures, such as intentions to seek help, we developed our own. To maximize the response proportion, we aimed to balance comprehensiveness with brevity, ensuring that surveys are clear, easy to interpret, and completable within 5 to 15 minutes.

Objective data on use metrics from Mindhelper will not be collected at an individual level, as this is not possible without violating the core principle of anonymity of Mindhelper users. However, data on the intervention group’s interactions with the SMS text message campaign will be collected at an aggregate level, and self-reported measures of use of Mindhelper will be collected in the surveys.

**Table 1:** Overview of measures and outcomes.

Measures and outcomes		Surveys			
		T1^a^	T2^b^	T3^c^	T4^d^
**Participant characteristics**					
	Age	✓			
	Gender (male, female, other)	✓			
	Primary occupation	✓			
	Educational level	✓			
	Parental educational level	✓			
	Region of residence	✓			
**Use of Mindhelper**					
	Digital behavior when feeling down	✓	✓	✓	✓
	Use of Mindhelper (in the intervention group)		✓	✓	✓
	Thoughts on the SMS text message campaign (in the intervention group)		✓	✓	✓
	Familiarity with or use of Mindhelper and Ro (at T1 for all participants and at T4 for the control group)	✓			✓
**Primary outcome**					
	World Health Organization–Five Well-Being Index	✓	✓	✓	✓
**Secondary outcome**					
	The Short Warwick-Edinburgh Mental Well-Being Scale	✓	✓	✓	✓
**Explorative outcome**					
	Intentions of help seeking	✓		✓	✓
	Barriers to help seeking	✓		✓	✓
**Other measures of mental health and well-being**					
	Patient Health Questionnaire-4	✓	✓	✓	✓
	The Short Form Perceived Stress Scale-4	✓		✓	✓
	Body Appreciation Scale-2 Short Form	✓	✓	✓	✓
	Diagnosed with a mental health issue	✓		✓	✓
	Affected by a diagnosis within the last month	✓		✓	✓
	Treated for diagnosis within the last month	✓		✓	✓
**Use of the health care system**					
	Absence from work or school	✓	✓	✓	✓
	Contact with the health care system	✓	✓	✓	✓

^a^T1: baseline.

^b^T2: 2 weeks after randomization.

^c^T3: 6 weeks after randomization.

^d^T4: 12 weeks after randomization.

#### Primary Outcome: the WHO-5

The WHO-5 captures positive aspects of mental health and well-being, aligning well with the promotion-focused objectives of Mindhelper. Furthermore, the measure is brief (5 items) and has demonstrated high sensitivity to changes in mental well-being, which is crucial when applied to an evaluation [36].

It consists of 5 positively framed items asking respondents to rate how often they felt cheerful, calm, active, rested, and interested in life over the past 2 weeks, using a 6-point Likert scale from 0 (not at all) to 5 (all the time). Scores range from 0 to 25; to standardize, the raw score is multiplied by 4, producing a final score from 0 to 100, where higher scores indicate greater well-being.

The WHO-5 is a widely recognized measure of mental well-being with extensive validation across diverse populations, including young people, and has been translated into more than 30 languages, including Danish [36,37]. Given its widespread use in mental health research, using the WHO-5 allows for comparability with other studies.

#### Secondary Outcome: The SWEMWBS

SWEMWBS, similar to WHO-5, is used to assess mental health and well-being by focusing on positive mental health aspects. It is a 7-item questionnaire, focusing on the past 2 weeks, covering aspects of both feeling good and functioning well, including optimism, relaxation, problem-solving, and interpersonal closeness. Each item is rated on a 5-point Likert scale from 1 (“none of the time”) to 5 (“all of the time”), with total scores indicating overall well-being; higher scores reflect greater well-being. Where the WHO-5 is an affective measure focusing on emotions and energy, the SWEMWBS is a hybrid measure that, in addition to measuring positive emotions, also includes aspects of functioning [38].

SWEMWBS is derived from the original 14-item version and is validated for screening mental well-being in both general and clinical populations, including young people, and has proven sensitive to changes [39].

#### Explorative Outcome: Intentions of Help Seeking

There is no standard approach for measuring intentions and barriers to help seeking, and existing studies have used a wide range of measures and definitions [5]. To retrieve a comprehensive measure of young people’s intentions of help seeking, we developed our own questions:

Imagine that you have been feeling very down over the past couple of months. Everything feels overwhelming, and you have started to feel that life is meaningless. How likely is it that you would seek help or support from the following if you found yourself in this situation? (1) Family, (2) Friends, (3) Partner, (4) A healthcare provider (e.g., doctor or psychologist), (5) Teacher, study counselor, manager, or HR., (6) Internet search (e.g. Google search or ChatGPT), (7) Social media (e.g., Instagram, TikTok and Reddit), (8) Telephone/chat counseling (e.g., Børnetelefonen or Headspace).

Response categories included “very likely,” “likely,” “unlikely,” “very unlikely,” and “don’t know/don’t have.”

The intention was to present the responders with a scenario that we consider to be of severity demanding support and help. Furthermore, a scenario related to symptoms of depression was chosen, as this is one of the most common issues among young people.

If participants indicate that they are unlikely or very unlikely to seek help from the listed sources of support, they are prompted to select their reasons from the following categories: (1) “I don’t think they can help me”; (2) “I prefer to handle it by myself”; (3) “I am afraid of burdening them”; (4) “I find it embarrassing/shameful to feel this way”; (5) “I have sought help but did not receive it”; (6) “I don’t believe I can get better”; or (7) “Other, please describe.” In addition, all participants are asked which factors might encourage them to seek help for mental health issues through an app or website and what barriers prevent them from doing so, explored through 4 different phrasings in open-text questions.

#### Other Measures of Mental Health and Well-Being

The Patient Health Questionnaire-4 (PHQ-4) is an ultrabrief self-report validated questionnaire that consists of a 2-item depression scale (Patient Health Questionnaire-2) and a 2-item anxiety scale (Generalized Anxiety Disorder-2). A validation study showed that the scale consists of 2 discrete factors (depression and anxiety) that explain 84% of the total variance [40]. An elevated PHQ-4 score is not a diagnostic tool but rather gives an indication of the presence of depression or anxiety.

The Short Form Perceived Stress Scale is an instrument for measuring perceived stress [41]. The Perceived Stress Scale-4 is designed to assess how unpredictable, uncontrollable, and overloaded respondents find their lives and provides a measure of an individual’s perceived stress levels.

The Body Appreciation Scale-2 (BAS-2) focuses on positive body image and not just body appearance. Since its release in 2015, the BAS-2 has been translated into more than 20 languages and is well-validated. BAS-2 consists of 10 items; however, the work by Tylka et al [42] demonstrated that 2 items explain more than 95% of the variance captured by the full 10-item questionnaire, named the BAS-2 Short Form. To shorten the survey, we decided to only include 2 items: “I feel love for my body” and “I am comfortable in my body.” We are using a Danish translation of the BAS-2, previously tested among people aged between 12 and 19 years [43]. BAS-2 has not yet been validated in a Danish context nor among people aged between 15 and 25 years; however, we are currently conducting this validation and will consider these results when interpreting our findings.

### Tests of Surveys and Setup

The initial survey was pilot-tested through cognitive interviews with 5 informants aged between 15 and 25 years. This process aimed to assess how the questions were interpreted and identify potential challenges in responding to the surveys [44]. Special attention was given to questions we had developed ourselves. On the basis of the informants’ feedback, the wording was adjusted for clarity, categories were refined to better capture expected answers, and some questions were omitted due to misinterpretations. In addition, as the preexisting scales for intentions of help seeking were found to be unfeasible, the researchers constructed a new scale for the study, inspired by preexisting scales and the informants’ comments. The constructed scale was tested among 4 new informants aged between 15 and 25 years and 2 from the initial test and adjusted accordingly.

Due to technical difficulties, an unplanned test phase of the survey setup was conducted. During this phase, 323 respondents, recruited via Facebook and Instagram, completed the T1 survey and were invited to participate in the follow-up surveys. The intervention group also received the SMS text message campaign invitation. However, technical errors led to some participants being invited for follow-up surveys at incorrect times, and most participants did not receive reminders for nonresponse. In addition, some participants in the intervention group received the SMS text message campaign invitation late or not at all. This phase allowed us to thoroughly assess the surveys. We reviewed all participants’ comments and corrected or omitted questions that did not work as intended.

### Sample Size Calculation

We performed a sample size calculation, considering an average effect size of 0.1 for mental well-being (on the WHO-5 scale) at T2 to be of public health relevance (it is equal to an improved WHO-5 score of 1.94 points on average in the intervention group compared to the control group). Even small effects on individual mental health outcomes can lead to a major impact at a population level, particularly when the service can be scaled widely and reach a large target audience.

We applied the conventional levels of statistical power (0.8) and level of significance (.05) and assumed an SD of 19.4 at T1 among the intervention and control groups combined (based on data from the feasibility study) [18]. On the basis of these assumptions, at least 3142 participants were required (n=1571, 50% in each group).

In the test phase, we retrieved a response proportion of 64.1% (323/504) at T2 (primary end point). Assuming a similar retention proportion, we aim to include 4910 participants. The proportion of participants responding to the follow-up surveys will be regularly reviewed to evaluate if the assumption regarding retention in the sample size calculation is correct or if the number of participants included should be increased or decreased.

### Planned Statistical Analysis

#### Overview

**Figure 3:**
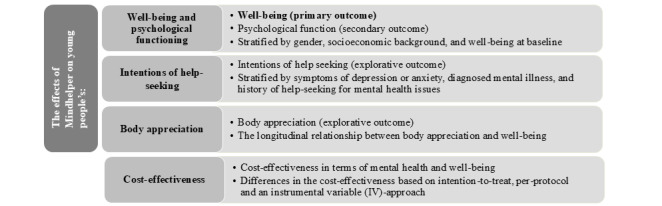
Overview of the planned analysis.

We will evaluate the effect of Mindhelper on multiple outcomes, as illustrated in Figure 3. First, we will conduct a randomization check and a dropout analysis. The primary analyses will follow the intention-to-treat principle, including all participants according to their randomized allocation, regardless of intervention use, and will be based on imputed data in cases of missingness. Missing data will be handled by multiple imputations using information from all the time points [45]. To test the robustness of the findings, sensitivity analyses will be conducted among complete cases. In addition, we will assess the complier average causal effect using the “contamination-adjusted intention-to-treat” method, an instrumental variable approach that accounts for adherence while preserving the benefits of randomization [46]. In this analysis, self-reported Mindhelper use at T4 in both the intervention and control groups will serve as the measure of adherence.

Analyses of data and interpretation of results will be done masked to the participant allocation. No interim analysis will be conducted during the trial. The subsequent section outlines the planned statistical analysis of each outcome and the cost-effectiveness analysis. A detailed statistical analysis plan has been published [47] before initiating any outcome analyses and unblinding the data.

#### The Effects of Mindhelper on Mental Well-Being and Psychological Functioning

The primary analysis will compare mean mental well-being, measured by WHO-5, between the intervention and control groups at T2, adjusted for WHO-5 scores at T1. If model assumptions are met, we will use a linear regression model. Psychological functioning, the secondary outcome measured by SWEMWBS, will be analyzed similarly as a continuous outcome, also adjusted for T1 values.

T2 was chosen for the primary assessment of effect because Mindhelper aims to provide immediate support and advice when problems arise. Most users visit thewebsite for a shorter period rather than regularly over multiple weeks. If the model assumptions are met, long-term effects will be evaluated at T3 and T4 using a repeated measures linear regression model.

To assess variation in effect by participants’ gender (male, female, or other) and socioeconomic background (parents’ highest educational attainment), analyses will be stratified accordingly. In addition, we will stratify by mental well-being at T1, expecting Mindhelper to be most effective for participants with moderate mental well-being (WHO-5 scores between 36 and 50) compared to those at high risk of depression or long-term stress (WHO-5 scores between 0 and 35) and those with mental well-being scores within the average range of the general population (WHO-5 scores >50). Furthermore, this stratified analysis will enable us to assess potential negative side effects of the intervention, assessing if the intervention group with WHO-5 scores >50 at T1 experienced a decline in mental well-being compared to the control group.

#### The Effects of Mindhelper on Intentions of Help Seeking

To evaluate the impact of Mindhelper on help-seeking intentions, we will compare scores on the constructed scale between the intervention and control group at T3, adjusted for T1 values. Potential long-term effects will be assessed at T4 using a model accounting for repeated measures. We selected T3 for this evaluation because we hypothesize that changes in help-seeking intentions take longer to manifest compared to the primary and secondary outcomes.

The 8 categories of support will be assessed separately. Furthermore, we will categorize the sources into 3 groups: informal sources (categories 1, 2, and 3), formal sources (categories 4, 5, and 8), and self-guided sources (categories 6 and 7). Special attention will be given to category 6, which is the most common way to access Mindhelper.

The severity of mental health issues at T1, along with previous help-seeking behaviors, may influence both the use of Mindhelper and its effect on help-seeking intentions. To account for this, analyses will be stratified by symptoms of depression and anxiety (PHQ-4) and intentions of help seeking at T1. Among those with a mental health diagnosis, we will further stratify by T1 levels of impairment by mental health diagnosis within the last month (daily, weekly, more seldom than weekly, not affected, or do not know) and whether any treatment was received within the past month for the diagnosis (treatment received, on waitlist for treatment, or no treatment received).

#### The Effect of Mindhelper on Body Appreciation

The effect of Mindhelper on body appreciation will be assessed by comparing body appreciation scores (measured using the BAS-2 Short Form [42]) between the intervention and control groups at T2, using a linear regression model (if model assumptions are met) and adjusted for T1 scores. If model assumptions are met, potential long-term effects will be evaluated at T3 and T4 using a repeated measures linear regression model.

In addition, we will explore the mediating role of mental well-being and psychological function (WHO-5 and SWEMWBS) in the relationship between Mindhelper and body appreciation. The mediation analysis will estimate the natural direct and indirect effects [48], providing insights into how mental well-being and psychological function may explain any influence of Mindhelper on body appreciation.

#### Cost-Effectiveness Analysis

The cost-effectiveness analysis will be performed by constructing a standard incremental cost-effectiveness ratio (ICER) expressing the additional cost per mean mental well-being score, measured by WHO-5, obtained from the randomization to Mindhelper compared with standard care [49]. The analysis will apply standard techniques from economic evaluation aimed at estimating costs per quality-adjusted life years gained. The results from this analysis will contribute to the evidence-based economic evaluation of interventions aimed at increasing mental health and well-being.

Incremental estimates of costs and mental well-being scores obtained will be assessed in a regression model. To examine the second-order uncertainty of the estimated ICERs, a probabilistic sensitivity analysis using a 5000 bootstrap resample with a 95% CI will be conducted. Cost-effectiveness planes illustrating the resampled ICERs and cost-effectiveness acceptability curves at T2 to T4 will be constructed to show the probability that the intervention is cost-effective given varying threshold values for an additional change in the mean mental well-being score obtained. No standardized fixed threshold for the value of an additional mean mental well-being score obtained from a new health intervention exists. Therefore, the probability of cost-effectiveness will be investigated for several thresholds, for example, values ranging from 0 to DKK 500,000 (US $78,038.43).

Both potential health care savings and direct costs of delivering the intervention will be included in the analysis. Information on self-reported all-cause demand for acute health care services, general practitioner visits, use, and absence from employment in the project period will be gathered from surveys. To operationalize this in monetary terms, the average unit cost of acute health care use and average hourly wage earnings depending on gender and age in Denmark will be used in the economic evaluation. For the intervention group, operating costs related to Mindhelper will also be included based on the overall cost per user. These costs cover development, maintenance, and technical support for the service. Cost estimates will be calculated based on list prices from health care providers, and scenarios of changes in the number of users will be explored.

### Transportability of Results

To assess how many young Danish people may benefit from Mindhelper, if it proves to be effective, we will compare the characteristics of our sample to a nationally representative sample of 31,420 young Danish people aged between 15 and 25 years. The national sample, drawn from the study “Young in 2023—Room for Everybody?” includes self-reported data on demographics, WHO-5, and PHQ-4 [50]. Furthermore, we will use the national sample to retrieve information on how our study population varies from a representative sample.

### Qualitative Contributions

To gain a deeper understanding of how young people use and integrate self-help tools from Mindhelper in their everyday lives to promote mental health, a qualitative interview study will be conducted. In total, 20 young people will be interviewed twice, approximately 3 months apart. Participants for the interviews are recruited from the intervention group at T3. From the pool of participants who provide consent to be contacted to participate in interviews, 20 participants will be selected, considering gender, age, region of residence, and medical history of mental health disorders, to ensure a diverse group. This information will be based on responses to the T1 survey and solely used for selection purposes without influencing the themes of the interviews.

Selected respondents will be contacted via SMS text messages to arrange a time for the first interview. After the first interview, respondents will again be contacted via SMS text messages to schedule the second interview. Upon completing both interviews, participants will receive a cinema gift card via an SMS text message. Each interview will last up to 1 hour and follow a semistructured interview guide. The interviews will be conducted by AOK via phone or Microsoft Teams or Zoom (Zoom Communications, Inc), based on the participant’s preference. The interviews will be audio recorded and transcribed verbatim. Conducting the first interview at T3 will allow for insights into how respondents experience receiving the intervention and how they have used it (eg, “What themes or tools on Mindhelper have you used?” “What did you gain from reading about these themes?” and “How has it been receiving the Mindhelper text messages?”). The second interview held after T4 will contribute to the knowledge of how respondents use Mindhelper following the SMS text message campaign’s end (eg, “Have you been on Mindhelper since you stopped receiving text messages about the website,” and “Has participating in this study changed your use of websites, apps, or social media to seek knowledge or advice about mental health?”). Themes for the first and second interviews are shown in Table 2. The qualitative data analysis software NVivo (Lumivero) will be used to process all data, with interviews analyzed according to the research questions, systematic text condensation, and template analysis [44,51].

**Table 2:** Themes for the first and second interviews.

Theme	First interview	Second interview
Background: gender, age, region of residence, and primary occupation	✓	
Knowledge of Mindhelper: How did they become aware of Mindhelper?	✓	
Use of Mindhelper: Have they used Mindhelper before or during the SMS text message campaign?	✓	
How they have used Mindhelper: What topics and features have they used on Mindhelper?	✓	✓
Use of Mindhelper after SMS text message campaign ended: Future use of Mindhelper?		✓
Impact of Mindhelper: What has the impact been? Are there any areas for improvement on Mindhelper?	✓	✓
Handling mental health struggles: What challenges does the young person face? What is their behavior in response to mental health struggles?	✓	
Handling mental health struggles: Are there any changes since last time?		✓
Digital behavior: How is the internet used in relation to mental health topics? Where do they get their information?	✓	
Help seeking—online versus network: When do they use the internet, and when do they turn to friends and family or professionals?	✓	

## Results

This study was fully funded in November 2022, and the data collection started in January 2025. As of August 2025, we have enrolled 2613 people. The data analysis will start after data collection concludes, by early 2026, and the results of the primary outcome are expected to be published in the fall of 2026.

## Discussion

### Anticipated Findings

This is the first large-scale RCT to evaluate the effects of a digital, multicomponent, self-guided mental health promotion service among young people. If effective, this highly scalable service may contribute to combating the trend of rising mental health issues among young people and address key challenges in primary care by delivering timely, coordinated, and effective services to young individuals, potentially at a low cost [52].

### Strengths and Limitations

This study has several strengths, including the large sample size, which enhances statistical power, enabling the detection of even small effect sizes that, while modest at the individual level, could generate substantial impact at the population level if the service is widely scalable and accessible. In addition, this study includes a more diverse participant sample than previous research, encompassing a broader population beyond specific university settings or patients with specific clinical symptom profiles [53,54]. This diversity may more accurately reflect the potential user base for the intervention. This is the first study to assess the cost-effectiveness of a digital, self-guided mental health promotion service targeting young people.

This study also has limitations, including that Mindhelper is publicly accessible and will continue to be so during the trial, raising the risk of exposure misclassification, which could dilute any observed differences between intervention and control groups. This misclassification would likely bias results toward the null, underestimating the intervention’s effect. However, in the feasibility study, very few control group participants accessed thewebsite [18]; therefore, we do not consider this to be a major risk.

### Conclusions

Individuals most commonly access Mindhelper via diagnostic and research-related search queries through search engines (eg, Google), where users are organically drawn to the content. In contrast, the Mindhelper study actively directs content to the intervention group. Therefore, our study deviates from the natural use patterns and evaluates the combined effects of the SMS text message campaign and Mindhelper. We create a situation that brings participants to Mindhelper, but Mindhelper and the use of Mindhelper are not designed for the intervention. Furthermore, conducting the study in Mindhelper’s natural use context would preclude the establishment of a control group, thereby making it impossible to evaluate its effects within the framework of an RCT.

A key challenge is expected to be adherence in the intervention group, meaning the extent to which participants in the intervention group engage with Mindhelper. Our feasibility study indicated (very) low engagement when participants were solely provided with a link to Mindhelper by the end of the T1 survey [18]. Therefore, the SMS text message campaign was developed, aiming to boost adherence; however, we do not know yet whether this was sufficient.

Low adherence could lead to misclassification of exposure within the intervention group, potentially introducing bias into results. If nonadherence is unrelated to mental health outcomes (nondifferential misclassification), this misclassification could reduce observed intervention effects. However, if nonadherence is related to mental health outcomes (differential misclassification), this could either inflate or attenuate the observed effect, depending on adherence patterns. We will conduct sensitivity analyses based on the as-treated principle (complier average causal effect) to assess potential effects under full adherence. Adherence data will be collected in binary format (yes or no) at T2 to T4 for the intervention group. For the control group, adherence will be assessed at T1 and T4. In the feasibility study, participants’ self-reports on specific Mindhelper features used did not align with their actual web history [18]. Therefore, we have revised our approach, asking the intervention group to describe how they have used Mindhelper and Ro apps in open-text questions, which will be coded with natural language processing models. Furthermore, qualitative information will provide insights into participant engagement and reasons for nonuse, and aggregated data on participants’ interaction with the SMS text message campaign will provide information on adherence.

It must be noted that the self-guided nature of Mindhelper and the diversity of topics covered mean that adherent participants in the intervention group may engage with different content and receive varying “doses” of the intervention, which aligns with the real-world application but makes the evaluation more complex.

Low retention rates are a major challenge in digital mental health trials [55]. In particular, for self-guided services, the average level of dropout from treatment for depression has been found to be 26%, while for guided services, it has been found to be 72% [56]. Retention proportion will be regularly reviewed to evaluate whether the assumption regarding retention in the sample size calculation is correct or whether the number of participants included should be revised to secure the statistical power of the primary analysis. Missing data will further be addressed through multiple imputations. If we retain a response proportion of 64% at T2 (primary end point), as was the case in the test phase of this study, this may not be as critical as in other studies of self-guided digital services. However, nonrandom dropout could still introduce bias into the findings, potentially skewing the results and overestimating or underestimating the true impact of Mindhelper, depending on the characteristics of those who drop out compared to those who remain in the study.

## Appendix

Multimedia Appendix 1 Themes and timing of the SMS campaign.

## Abbreviations

BAS-2: Body Appreciation Scale-2
ICER: incremental cost-effectiveness ratio
PHQ-4: Patient Health Questionnaire-4
RCT: randomized controlled trial
SWEMWBS: Short Warwick-Edinburgh Mental Well-Being Scale
T1: baseline
T2: 2 weeks after randomization
T3: 6 weeks after randomization
T4: 12 weeks after randomization
WHO-5: World Health Organization–Five Well-Being Index
