# Supplementary material for: A Self-Guided Digital Mental Health Promotion Service Targeting Young People: Protocol for a Randomized Controlled Trial
Source: JMIR Res Protoc. 2025 Sep 10;14:e73736. doi: 10.2196/73736 (PMC12461159; doi:10.2196/73736)
Supplement: Multimedia Appendix 1 [file resprot_v14i1e73736_app1.docx]

**Appendix 1: Themes and timing of the SMS campaign.**

| **No** | **Theme** | **Distribution time** |
| --- | --- | --- |
|  | Start of study | Day 0: Be assigned to the intervention group after completing the baseline survey. |
| 1 | Welcome | Day 1 |
| 2 | The alternate breath | Day 3 |
| 3 | Gratitude list | Day 5 |
| 4 | Online course on anxiety | Day 7 |
| 5 | Self-care | Day 9 |
| 6 | Meet Amalie and Victor from the letterbox | Day 11 |
| 7 | Ro: an app for stress | Day 13 |
|  | 2. survey distributed | Day 14 |
|  | Reminder to respond to 2. survey | Day 15 |
|  | Second reminder to respond to 2. survey | Day 16 |
| 8 | Falling a sleep | Day 17 |
| 9 | Worries | Day 20 |
| 10 | End the day by focusing on the good things in your life | Day 23 |
| 11 | Box Breathing | Day 26 |
| 12 | You are not alone if you are having a hard time | Day 29 |
| 13 | Mindfulness course | Day 32 |
| 14 | Share your problems with others | Day 35 |
| 15 | Halfway there, a high-five from Linn | Day 38 |
| 16 | Write a letter of gratitude to someone you appreciate | Day 41 |
|  | 3. survey distributed | Day 42 |
|  | Reminder to respond to 3. survey | Day 43 |
|  | Second reminder to respond to 3. survey | Day 44 |
| 17 | Conversation cards about friendship | Day 45 |
| 18 | Get to know new people | Day 48 |
| 19 | Nervousness | Day 51 |
| 20 | Pomodoro (Difficulty concentrating) | Day 54 |
| 21 | Honey depots | Day 57 |
| 22 | Course on prioritization and planning | Day 60 |
| 23 | Discontent - gratitude | Day 63 |
| 24 | Online course on anxiety (also presented on day 7) | Day 66 |
| 25 | Three mini-meditations | Day 70 |
| 26 | Stress | Day 74 |
| 27 | Be kind to yourself when life is hard | Day 78 |
| 28 | Shake tension out of the body | Day 81 |
| 29 | Thank you for now | Day 83 |
|  | 4. survey distributed | Day 84 |
|  | Reminder to respond to 4. survey | Day 85 |
|  | Second reminder to respond to 4. survey | Day 86 |
| End of study | | |
